# Fetal valproate syndrome in a 2-month-old male infant

**DOI:** 10.4103/0256-4947.62839

**Published:** 2010

**Authors:** Syed A. Zaki, Amol Phulsundar, Preeti Shanbag, Anupama Mauskar

**Affiliations:** From the Department of Pediatrics, Lokmanya Tilak Municipal General Hospital, Mumbai, India

## Abstract

Fetal valproate syndrome (FVS) results from prenatal exposure to valproic acid. It is characterized by a distinctive facial appearance, a cluster of minor and major anomalies and central nervous system dysfunction. We describe a 2-month-old male infant with the typical dysmorphic features characteristic of FVS. He had a persistent left superior vena cava draining into a dilated coronary sinus and mild pulmonary hypertension. There was a history of maternal intake of sodium valproate during pregnancy.

Valproic acid (VPA) is a widely used antiepileptic drug and mood stabilizer. It was first introduced for use as an antiepileptic drug in 1964 and is still a commonly used antiepileptic drug (AED) worldwide.^1^ A description of the teratogenic effect of the drug was first published in 1980.^1^ Since then many potential teratogenic and dysmorphogenic effects of VPA have been reported. We report a case of fetal valproate syndrome (FVS) in a 2-month-old male infant born of an epileptic mother who was taking sodium valproate during pregnancy.

## CASE

A 2-month-old male infant was brought to our outpatient department for evaluation of bilateral inguinal hernia. He was the first child born of a non-consanguineous marriage. His mother had been taking sodium valproate for epilepsy for the past 12 years and was on a dose of 1200 mg/day throughout her pregnancy. The infant was born at full term at a peripheral hospital by emergency by cesarean delivery, the indication being fetal distress. His birth weight was 2 kg. The baby cried immediately after birth. He was admitted to the NICU for respiratory distress and was discharged on breast feeds on the third postnatal day. The intrauterine growth retardation and the teratogenic effects of the antiepileptic drug were not considered by the health care providers at the peripheral hospital. Hence, no specific tests were done in the antenatal period.

**Figure 1:**
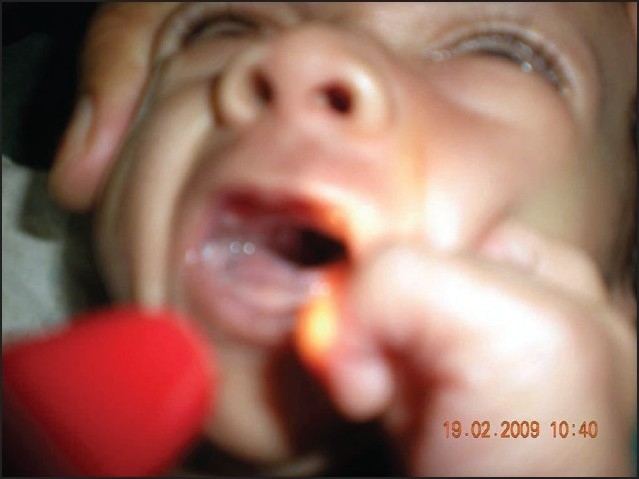
Examination of the oral cavity showing isolated cleft of the secondary palate.

**Figure 2:**
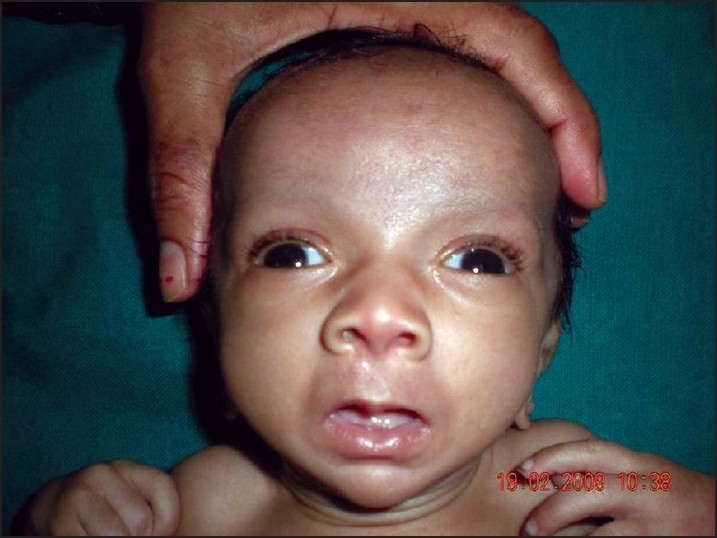
Note low set ears, broad and depressed nasal bridge, long philtrum, upturned nose, thin upper lip, thick lower lip, thin vermillion border, small mouth, and medial deficiency of eyebrows.

**Figure 3:**
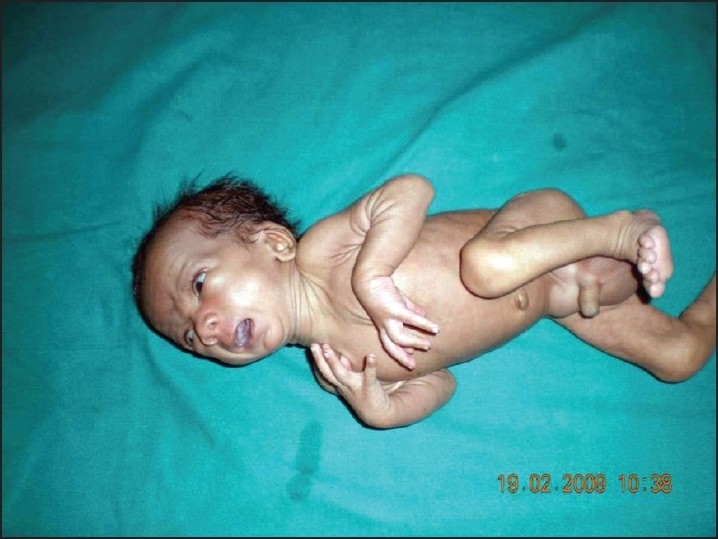
Bilateral radial club hand and broad hands, loose skin, bilateral inguinal hernia.

On examination at our hospital, his weight was 1.5 kg and length 52 cm (below 5th centile for age and sex) suggestive of failure to thrive. On enquiry the mother gave a history of nasal regurgitation of milk since birth. Examination of the oral cavity revealed the presence of a cleft in the soft palate (Figure 1). Dysmorphic features were present in the form of low-set ears, a broad and depressed nasal bridge, long philtrum, upturned nose, thin upper lip, thick lower lip, thin vermillion borders, small mouth, and medial deficiency of eyebrows (Figure 2). Broad hands and feet, bilateral radial club hand, loose skin, bilateral inguinal hernia (Figure 3) were other features.

Two-dimensional echocardiography showed a persistent left superior vena cava draining into a dilated coronary sinus and mild pulmonary hypertension. Ultrasound of the abdomen showed no abnormality. With a history of maternal intake of sodium valproate during pregnancy and typical dysmorphic features, a diagnosis of fetal valproate syndrome (FVS) was made.

## DISCUSSION

None of the AEDs available currently are completely safe during pregnancy, but VPA appears to be the most teratogenic. In a study done by Morrow et al on the major congenital malformation (MCM) risks of antiepileptic drugs in pregnancy, the overall risk for all AED exposed cases was 4.2%. The MCM rate was higher for polytherapy than for monotherapy. Polytherapy regimens containing valproate had significantly more MCMs than those not containing valproate. For monotherapy exposures, carbamazepine was associated with the lowest risk of MCM and those exposed to more than 1000 mg of valproate had the highest MCM rate than for any other monotherapy exposure.^2^

There is also a controversy as to whether epilepsy itself contributes to antiepileptic drug teratogenecity, thus confounding the teratogenic risk of a drug used for its treatment. However VPA, and not the underlying epilepsy syndromes, has been found to be associated with the elevated risk for malformations in the drug exposed fetus.^3^ VPA is associated with the highest risk of birth defects, especially with doses exceeding 1000 mg/day.^4^ Various factors contribute to the teratogenecity of VPA. These include the number of antiepileptic drugs that are co-administered, drug dosage, differences in maternal and/or infant metabolism and the gestational age of the fetus at exposure.^5^ VPA crosses the placenta and is present in a higher concentration in the fetus than in the mother. There is a 6 to 7 times increased risk of congenital malformations in babies of mothers exposed to valproate. Major congenital malformations are neural tube defects, congenital heart defects, oral clefts, genital abnormalities and limb defects. Other less frequent abnormalities include inguinal and umblical hernia, supernumerary nipple, postaxial polydactyly, bifid ribs and pre-axial defects of the feet.^6^ Zinc deficiency has been suggested as a possible cause of neural tube defects associated with valproate exposure as sodium valproate readily binds to zinc.^7^ Our patient had the typical facial features, bilateral inguinal hernia, low birth weight, upper limb and cardiac malformation. A distinctive facial phenotype of fetal valproate syndrome has been described which tends to evolve with age. The facial features are tall forehead with bifrontal narrowing, medial deficiency of eyebrows, infraorbital groove, trigonocephaly, flat nasal bridge, broad nasal root, antiverted nares, shallow philtrum, epicanthic folds, long upper lip with thin vermillion borders, thick lower lip and small downturned mouth.^5^

There is a 10 times increased risk of neural tube defects in babies of mothers exposed to VPA. The risk is maximum with VPA therapy, as compared to other anticonvulsants. The incidence of congenital heart disease is estimated to be around 4 times than that seen in the general population.^5^ Aortic valve stenosis, secundum atrial septal defect, pulmonary atresia without ventricular septal defect, perimembranous ventricular septal defect, interrupted aortic arch, hypoplastic left heart syndrome septal defects and valvular problems are known to occur.^6^ Our case had left superior vena cava opening into a dilated coronary sinus.

Oral clefts are 5 times more frequent than expected. Limb defects, digital abnormalities of various types and minor skin defects are known to occur.^5^ The association of developmental delay with VPA exposure has also been described.^5^ Pregnancy usually proceeds uneventfully and 10% of babies are small-for-gestational age as in our patient. Postnatal growth appears to be normal and general health is good.^3^ Microcephaly tends to occur only in the infants who are also exposed to other anticonvulsants. Prenatal diagnosis is focused on the detection of neural tube defects (NTD), as they are the proven major malformations. Estimation of maternal serum alpha-fetoprotein (AFP) can be used as a screening test for the presence of open NTD.

During pregnancy antiepileptic drugs should be administered as monotherapy and in the lowest possible dose with constant monitoring of serum concentration of antiepileptic drugs.^8^ VPA should not be routinely prescribed to women of child-bearing potential. If there is no effective alternative, then doses should be limited to a maximum of 1 gram per day, administered in divided doses and in the slow release form. Also high dose folic acid (5 mg/day) is recommended during pregnancy, starting at least 6 weeks pre-conception and continuing through the first trimester.^8^

The efficacy of VPA as an AED cannot be disputed, but the extent of its teratogenic effects cannot be under-estimated either. Hence, a balance between the therapeutic effects of this drug and its teratogenic effects is critical in the management of pregnant women with epilepsy.
